# Female vulnerability to the development of depression-like behavior in a rat model of intimate partner violence is related to anxious temperament, coping responses, and amygdala vasopressin receptor 1a expression

**DOI:** 10.3389/fnbeh.2013.00035

**Published:** 2013-05-01

**Authors:** G. L. Poirier, M. I. Cordero, C. Sandi

**Affiliations:** ^1^Laboratory of Behavioral Genetics, École Polytechnique Fédérale de Lausanne, Brain Mind InstituteLausanne, Switzerland; ^2^Child and Adolescent Service of Psychiatry, Hospital University of GenevaGeneva, Switzerland

**Keywords:** vulnerability indicators, resilience indicators, domestic violence, vasopressin receptor subtype 1a, serotonin, anxiety, social stress, individual differences

## Abstract

Exposure to violence is traumatic and an important source of mental health disturbance, yet the factors associated with victimization remain incompletely understood. The aim of the present study was to investigate factors related to vulnerability to depression-like behaviors in females. An animal model of intimate partner violence, which was previously shown to produce long-lasting behavioral effects in females as a result of male partner aggression, was used. The associations among the degree of partner aggression, the long-term consequences on depressive-like behavior, and the impact of the anxious temperament of the female were examined. In a separate group, pre-selected neural markers were evaluated in the amygdala and the lateral septum of females. Expression was examined by analyses of targeted candidate genes, serotonin transporter (*slc6a4*), vasopressin receptor 1a, (*avpr1a*), and oxytocin receptor (*oxtr*). Structural equation modeling revealed that the female's temperament moderated depressive-like behavior that was induced by cohabitation aggression from the male partner. More specifically, increased floating in the forced swim test following male aggression was most apparent in females exhibiting more anxiety-like behavior (i.e., less open arm exploration in an elevated plus-maze) prior to the cohabitation. Aggression reduced *slc6a4* levels in the lateral septum. However, the interaction between partner aggression and the anxious temperament of the female affected the expression of *avpr1a* in the amygdala. Although, aggression reduced levels of this marker in females with high anxiety, no such pattern was observed in females with low anxiety. These results identify important characteristics in females that moderate the impact of male aggression. Furthermore, these results provide potential therapeutic targets of interest in the amygdala and the lateral septum to help improve post-stress behavioral pathology and increase resilience to social adversity.

## Introduction

It has long been recognized that individuals who are exposed to adverse situations present with varying psychopathological outcomes, depending on factors such as individual characteristics and socio-environmental milieu that may help buffer the effects of adversity. These factors have been captured in a variety of bio-psycho-social models that have flourished in recent years.

Intimate partner violence is one of the most common forms of violence against women (Watts and Zimmerman, 2002). It is often associated with chronic post-traumatic stress disorder and depressive outcomes (Beydoun et al., 2012), both of which are related to the intensity of aggression by the partner (Cascardi et al., 1999). However, little is known about the underlying mechanisms that confer vulnerability to lasting emotional consequences. The main aims of the present report are to identify neural alterations that accompany symptoms caused by cohabitation with an aggressive partner and to determine whether anxious temperament may play a role in determining the consequences of such social adversity.

A critical development in our understanding of the consequences of stressful experiences is the recognition that one's interpretation of and coping with an experience may be more important than the event itself in determining the individual's psychopathological outcome (Lazarus and Folkman, 1984). Several predisposing vulnerabilities to a negative psychological outcome have been identified, comprising numerous and diverse aspects of cognitive and emotional styles (e.g., reviewed in Elwood et al., 2009). Anxiety-related aspects appear to play an important role in the psychopathological outcome. For example, high anxiety interacts with trauma exposure in the elicitation of anxiety-related distress (Larsson et al., 2008). Among women, anxiety sensitivity has been found to interact with negative life events, predicting increased posttraumatic stress symptoms (Feldner et al., 2008), particularly dysphoria (Elwood et al., 2009).

Perpetrator characteristics appear to incompletely explain the negative interactions and the clinical psychopathological outcome in human couples. For example, negative emotionality, including anxious reactions, can be associated with not only perpetration of abuse but also victimization (Moffitt et al., 2001; Robins et al., 2002). Emphasizing the dyadic nature of human relationships, Moffitt et al. (2001) suggested that both partners need to be taken into account to understand processes that define relationship quality, and ultimately, to improve prevention and treatment of psychopathological outcomes. Mechanisms underlying this dyadic relationship are further examined here.

A rat model of intimate partner violence, based on male exposure to peripubertal stress, was recently introduced by our laboratory (Cordero et al., 2012; Márquez et al., 2013). In investigating the neural mechanisms involved in exposure to acts of aggression in a controlled fashion, this model may help elucidate the vulnerabilities to psychopathological outcomes in victims of intimate partner violence, who are arguably relatively less studied than perpetrators. A further benefit of this rat model is the absence of assortative mating, a common issue in human studies and a potential confound for understanding causal events in this dyadic relationship (e.g., Frisell et al., 2012).

We have previously reported that in this model, females exposed to more aggressive partners developed behaviors that were reminiscent of abused and depressed women (Cordero et al., 2012). This pattern was accompanied by alterations in the dorsal raphe nucleus (Cordero et al., 2012), the main source of serotonin and a critical region in emotionality (Lowry et al., 2005). Here, in the context of an aggressive experience, we sought to examine the hypothesis that an anxious temperament would increase the level of depressive-like behavior induced by the stressful experience (Sandi and Richter-Levin, 2009) by affecting coping behaviors. In order to further examine the molecular substrates of resilience and vulnerability to depressive-like symptoms, we also investigated potential changes in gene expression in selected targets of the dorsal raphe nucleus, namely, the amygdala, and the lateral septum. The amygdala is a key node in the neural circuit that is engaged by fear and threat assessment (Mahan and Ressler, 2012), and the lateral septum is commonly associated with emotionality and social behaviors, including affiliative responses in humans (Sheehan et al., 2004; Moll et al., 2012). Candidate genes of interest implicated in depression and social affect were examined, including the serotonin transporter *slc6a4* and receptors for the neuropeptides oxytocin (*oxtr*) and vasopressin [the main receptor, *avpr1a*; for reviews, see Beck (2008); Neumann and Landgraf (2012)]. Overall, this work examines how individual differences in anxious temperament among females relate to gene activity in the brain with respect to resilience to the long-term effects of aggression victimization.

## Materials and methods

In order to address issues of resilience, novel analyses of unpublished data from an earlier study using our intimate partner violence model (Cordero et al., 2012) are presented here, accompanied by a new cohort of subjects for neural marker assays. The methods for the intimate partner violence model and accompanying analyses have been previously published in detail (Cordero et al., 2012).

### Subjects

Wistar Han rats were obtained from Charles River Laboratories (Lyon, France). Animals were maintained under controlled conditions (12-h light/dark cycle; lights on at 7:00 a.m.; 22 ± 2°C). Food and water were available *ad libitum*. With the exception of the home cage interactions (see details below), animal testing occurred during the first half of the animals' light phase. All procedures conformed to the Swiss National Institutional Guidelines on Animal Experimentation and were approved through a license by the Swiss Cantonal Veterinary Office Committee for Animal Experimentation.

Adult female virgin rats (12 weeks old) were screened for anxiety-like behavior using the elevated plus-maze (Pellow et al., 1985), a test that is widely used to evaluate animals' anxiety-related behaviors. Females were subsequently assigned to a male according to their weight, in order to preclude pairings with larger size differences that could affect the male–female interaction. After 21 days of cohabitation with a male, subjects either (1), after parturition and weaning, were housed in groups of 3 for 1 week and behaviorally characterized for lasting consequences on emotionality (*n* = 41), as indicated by the depressive-like behavior measured in the forced swim paradigm (see details below), or (2) were processed for brain extraction (*n* = 24; see details below).

### Elevated plus maze

The elevated plus-maze consists of two opposing open arms (each one measuring 45 × 10 cm) and two closed arms (each one measuring 45 × 10 × 50 cm) that extend from a central platform (10 × 10 cm) that is elevated 65 cm above the floor. The rats were placed individually on the central platform, always facing the same enclosed arm, and were allowed to freely explore the maze for 5 min. The parameters that were evaluated with the video tracking system (Ethovision 3.1.16, Noldus, Wageningen, Netherlands) were the total distance traveled (cm) and time spent (s) in the open and closed arms, the frequency of entries into each type of arm, and the velocity (cm/s). The floor of the apparatus was washed after each test with a 1% acetic acid solution to remove odors left by the previous subject. The anxiety-like behavior used to assign females in an unbiased fashion to the stress groups in the previous report (Cordero et al., 2012) was measured as the percentage of time spent in the open arms of the elevated plus-maze in a test conducted prior to cohabitation.

### Male–female cohabitation

Adult male virgin rats (12 weeks old) each cohabitated with a female. The focus here is on absolute levels of aggression directed at the female, and since in the previous study (Cordero et al., 2012) subjects in each group, control, and peripubertally stressed, exhibited some aggressive behaviors, presently no distinction is made regarding the origins of the male behavior, which will not be further discussed.

During male–female cohabitation, the home cage was changed three times (once per week) at approximately 17–19 h. The cage change is an arousing experience that stimulates social interactions, and for this reason, it was used as the starting time for behavioral observations. At 1-week intervals, immediately upon entering each new fresh cage, social interactions were video recorded for 30min. The duration of attacks, lateral threats, upright, and keep down behaviors exhibited by the male toward the female, and the time during which the female displayed defensive-submissive behavior (either freezing or being in a supine position under the male) were scored by an experimenter who was blinded to the treatment conditions and assisted by a computer program (The Observer 5.0.25, Noldus, Wageningen, Netherlands). The measure of aggression is the summed percentage of attacks, lateral threats, upright, and keep down behaviors exhibited by the male partner. Aggression and female defensive-submissive behaviors were averaged across the three cohabitation week samples. Animals were not visibly wounded by these behaviors, except for superficial scars on a few females.

### Forced swim test

For this test, we used an adapted version of the original rat forced swim test (Porsolt et al., 1978), in which a passive, floating behavioral response is thought to indicate depressive-like behavior. Rats were individually placed for 15 min in a plastic beaker (25 cm diameter, 46 cm deep) filled with water (25 ± 1°C) to a height of 30 cm. The rats were then removed from the tank, gently dried with a towel, returned to their home cages, and then returned to the water 24 h later for 5 min. The total duration of floating was measured for each forced swim test session. Rats were considered to show floating (immobility) behavior when they did not struggle, only making the movements necessary to keep their heads above water. The water was changed after each session, and the cylinder was cleaned to avoid the influence of alarm pheromones that were left behind by the previous animal. For the present purpose, for a pure measure of floating, we analyzed the behavior of the rats on the first day of the forced swim test, since the rats had experienced prior stress that can facilitate immobility, without the need for a water pre-exposure (Borsini et al., 1989).

### Gene expression

**Figure 1:**
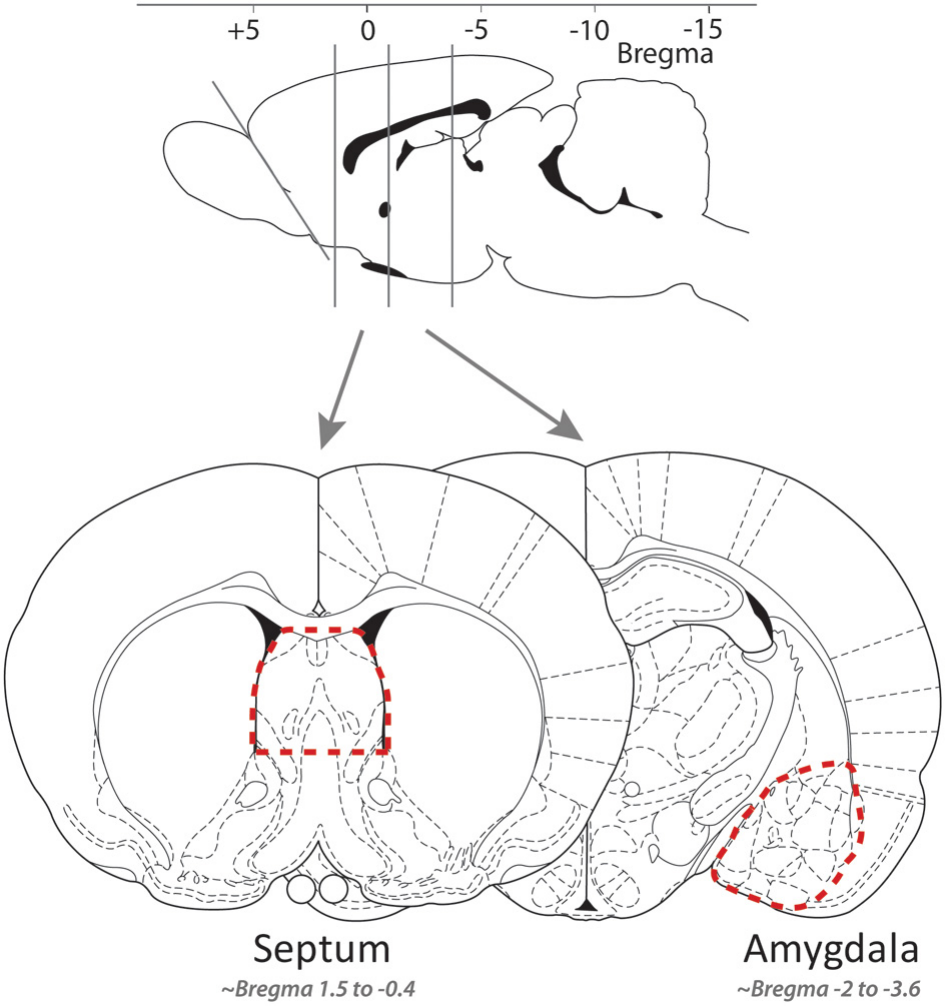
**The approximate location of the coronal slices along the rostro-caudal axis (top) and of the tissue isolated for the lateral septum and amygdala samples [bottom; adapted from Paxinos and Watson (1997)]**.

Fresh brains were removed, and over ice coronal slices produced with a razor blade, the lateral septum and the extended amygdala quickly dissected using fine curved forceps (slices respectively approximately −0.4 to 1.6 and −3.6 to 2.12 mm from bregma, *cf*. Figure 1; Paxinos and Watson, 1997). Tissue samples were quickly placed in RNAse free cryotubes, flash-frozen in liquid nitrogen, and stored at −80°C until further processing. RNA was isolated using the RNAqueous-Micro kit (Ambion, Applied Biosystems, Rotkreuz, Switzerland). Following ethanol precipitation and quantification with Nanodrop (Thermo Fisher Scientific, Wohlen, Switzerland), cDNA was synthesized using the Superscript VILO kit (Invitrogen, Basel, Switzerland), and quantitative real-time PCR reactions (Applied Biosystems 7900HT) were conducted in triplicate using Power SYBR Green PCR Master Mix and primers for *slc6a4*, *avpr1a*, and *oxtr* designed to be complementary to each gene of interest [Microsynth (Balgach, Switzerland; see sequences in Table 1)]. Gene expression was normalized to the internal ribosomal reference genes *rps-18* and *rps-29*, and the analyses were conducted with qBase 1.3.5 (Hellemans et al., 2007) using the comparative cycle threshold method, yielding [delta][delta]Ct = [delta]Ct,sample—[delta]Ct,reference. The efficiency of all of the primer pairs was confirmed by performing reactions with serially diluted samples. The specificity of all of the primer pairs was confirmed by analyzing the dissociation curve.

**Table 1:** **Primer sequences for the gene expression analyses of the selected candidates**.

*slc6a4* F	AAACGGGTGCATTTCCATATG
*slc6a4* R	GGCGTAACCAATGCCTTTGA
*avpr1a* F	AACGAACAGCACTGGGATGTG
*avpr1a* R	GGAATGAATCTGATGGATTTGGAA
*oxtr* F	CATCACCTTCCGCTTCTATGG
*oxtr* R	ATGCCCACCACCTGCAAGTA
*rps-18* F	TCATGCAGAACCCACGACAA
*rps-18* R	TCACGTCCTTCTGTCTGTTCAAG
*rps-29* F	GCCGCGTCTGCTCTAACC
*rps-29* R	GCACATGTTCAGCCCGTATTT

F, forward; R, reverse.

### Statistical analyses

#### Behavior

Bivariate correlations, Mann–Whitney non-parametric comparisons, Student *t*-tests, and univariate analyses of variance (ANOVA) were conducted using SPSS (Statistical Package for the Social Sciences) software (Zürich, Switzerland). Preliminary analyses were conducted by producing “low”and “high” subgroups based on a median split of behavioral measures, examining male aggression and female elevated plus-maze exploration (all subjects were included).

Using a structural equation modeling approach, an interactional model was applied (Amos 17.0, SPSS, Zürich, Switzerland). Multivariate approaches decompose the variable relationships into separate components by *concurrently* accounting for each variable under investigation (unlike bivariate analyses). The form of the model tested examined how characteristics of each subject in the dyad interacted to determine the consequences for the female. Specifically, we examined whether female anxiety-like behavior moderated the effect of partner aggression on defensive-submissive behavior and whether those variables together determined the depressive-like outcome. For this moderation analysis, behavioral measures were treated as continuous rather than discrete, categorical variables because of the greater statistical power that this approach provides (cf. Lazic, 2008). Because of their degree of distribution non-normality, a square root transformation was applied to partner aggression and female defensive-submissive values (Tabachnik and Fidell, 1996). Data for the independent (predictor) and the proposed moderating variables were standardized [*Z*-score; as recommended in Frazier et al. (2004)], and an interaction term was obtained by calculating their product. In the model, a significant relationship between the interaction term and the dependent variable indicates moderation. Covariances between each open arm exploration and male aggression with their product-term were omitted after observing their non-significance, and the final model that was tested is presented. Model fit indices were the comparative fit index (CFI), the root mean square error of approximation (RMSEA), and χ^2^. Typically, a good-fitting model that is a plausible representation of the underlying data structure is expected to have a non-significant χ^2^, CFI ≥ 0.90–0.95 and RMSEA < 0.05 (*p* close > 0.05; Tabachnik and Fidell, 1996). Finally, the moderation was plotted using the Stats Tool Package, with values ±1 standard deviation from the mean representing low vs. high levels (Gaskin, 2012).

#### Gene expression

For univariate gene expression analyses, expression values were normalized to the control group to visualize the fold change. For Two-Way analyses of variance, subgroups were identified according to a median split on behavioral measures, examining male aggression (relatively “low” vs. “high”) and elevated plus-maze exploration (relatively “low” vs. “high”; all subjects were thus included).

## Results

### Behavioral patterns associated with the depressive-like outcome of experiencing long-term aggression

**Figure 2:**
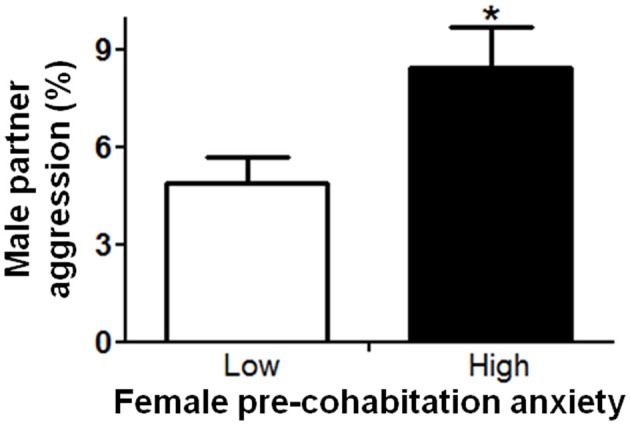
**Female anxiety-like behavior, as observed on the elevated plus-maze prior to cohabitation, was associated with increased aggression by the male partner.** The mean ± standard error of the mean are presented. ^*^p < 0.05.

**Figure 3:**
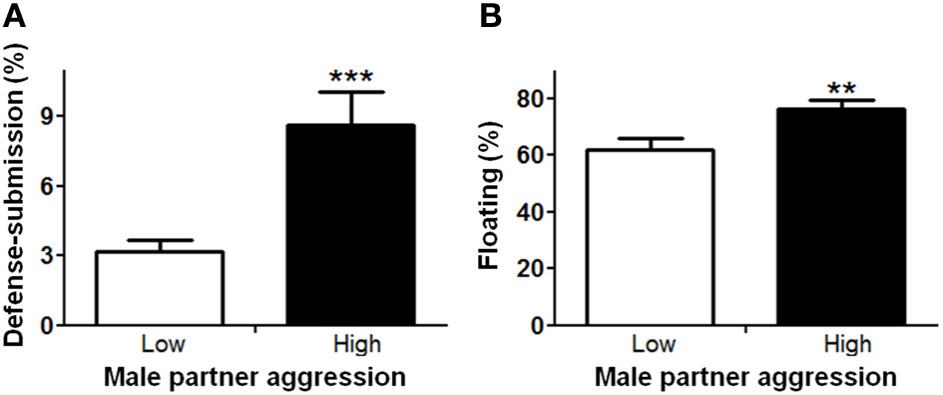
**Higher aggression by the male partner during cohabitation significantly increased (A) defensive-submissive behaviors and (B) depressive-like behavior, as determined by floating behavior.** The mean ± standard error of the mean are presented. ^**^p < 0.01; ^***^p < 0.001.

In order to understand factors conferring resiliency to the development of depressive-like symptomatology, we examined whether male aggression elicited female defensive-submissive behavior and whether, the temperament of the female prior to cohabitation played a role in the elicitation of such behavior. Preliminary analyses were based on subgroups of female anxiety-like behavior, operationally defined as less exploration of the open arms of the plus-maze (“low” vs. “high,” mean percent (±S.E.M.) = 22.3(±1.8) and 6.2(±0.8), with *n* = 20 and 21, respectively; *U* = 0, exact *p* < 0.001). First, female anxiety-like behavior, was found to be associated with enhanced aggression by the male partner (Figure 2; *U* = 123.0, exact *p* < 0.05). Second, partner aggression (“low” vs. “high”, mean percent (±S.E.M.) = 3.0(±0.3) and 10.3(±1.0), with *n* = 20 and 21, respectively; *U* = 0, exact *p* < 0.001), as expected, elicited defensive-submissive behavior in the females (Figure 3A; *U* = 79.0, exact *p* < 0.001) and led to increased depressive-like behavior, as measured by the observation of floating by the females in the forced swim paradigm [Figure 3B; *t*_(39)_ = −2.86, *p* < 0.01].

We next proceeded to examine the relationships among all of these variables concurrently using a structural modeling approach. As a usual first step with this approach, we present the correlations among female anxiety-like behavior, male partner aggression, female defensive-submissive behavior, and female depressive-like behaviors (Table 2). We found that aggression by the male partner was significantly negatively correlated with the percentage of time spent in the elevated plus-maze open arms before cohabitation. Aggression by the male partner was also, as one would expect, significantly positively correlated with female defensive behavior and with the extent of floating behavior.

**Table 2:** **Bivariate correlations for the behavioral measures used in the model**.

		**EPM open arm (% pre-cohabitation)**	**Partner aggression (√%)**	**Defensive-submissive behavior (√%)**	**Floating (%)**
EPM open arm (% pre-cohabitation)	*r*	–	−0.44^**^	−0.10	−0.23
	*p*		*0.004*	*0.546*	*0.150*
Partner aggression (√%)	*r*		–	0.48^**^	0.34^*^
	*p*			*0.001*	*0.030*
Defensive-submissive behavior (√%)	*r*			–	−0.16
	*p*				*0.327*
Floating (%)	*r*				–
	*p*				

EPM, elevated plus-maze; r, Pearson correlation (values in bold font when significant); p, two-tailed statistical significance (italics); significant p-values,

* p < 0.05;

** p < 0.01; n = 41.

In contrast, none of the correlations were significant between any of the open arm, female defensive, or floating behaviors, as shown in Table 2. Therefore, these correlational data suggest that female temperament and defensive behaviors correlate independently with male aggressive behavior. Therefore, female temperament and defensive behaviors could be considered separate factors, according to this correlational analysis.

### Modeling the behavioral factors conferring resilience to depressive-like behavior

**Figure 4:**
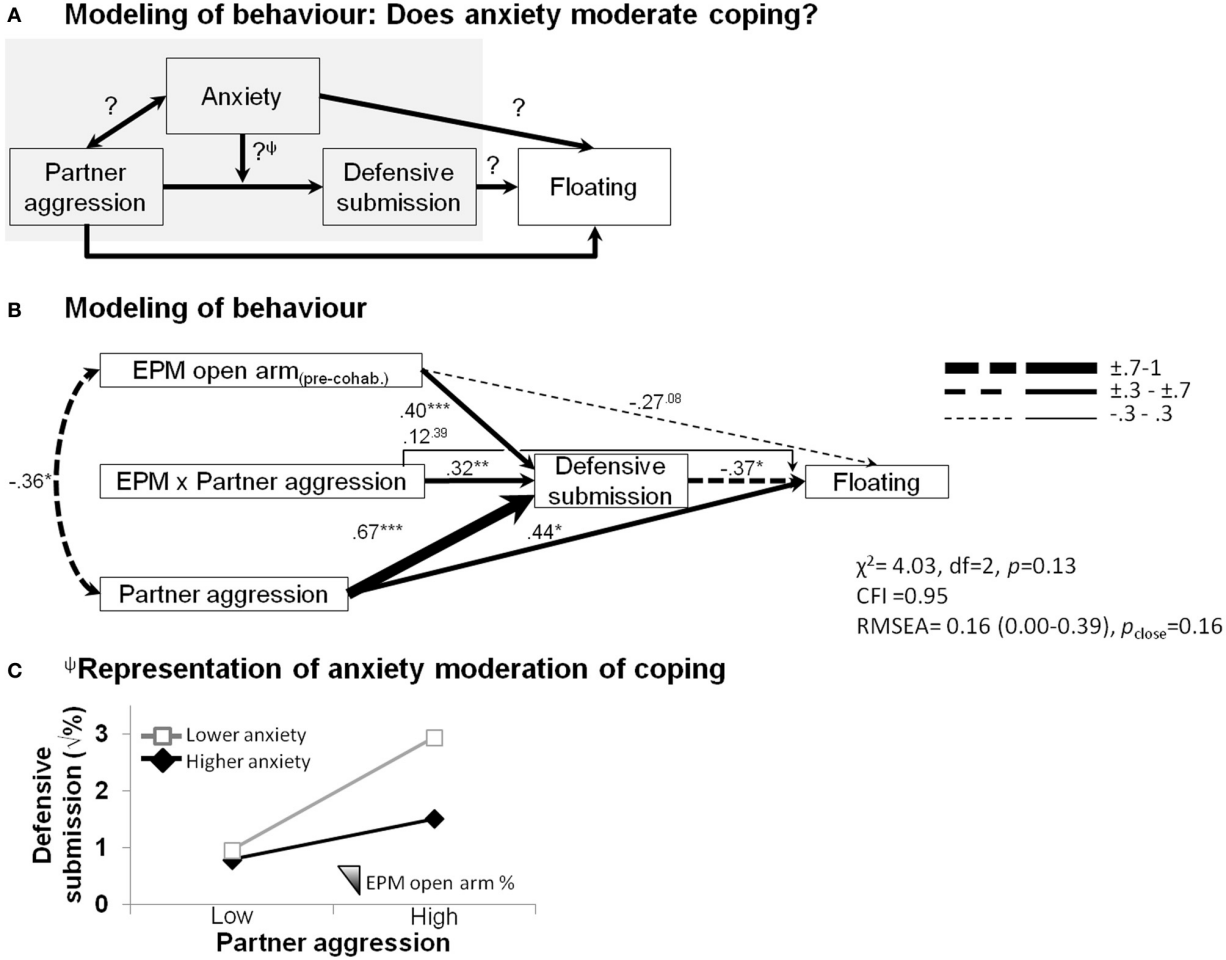
**Structural equation modeling of behaviors of each partner predicting female depressive-like outcome.** Anxiety moderates the impact of partner aggression on the development of depressive-like floating behavior. **(A)** Integration of the behavioral patterns using structural equation modeling. Note that the result of the moderation analysis^Ψ^ is graphically presented in **(C)**. **(B)** Partner aggression (1) predicted more floating, a depressive-like outcome; (2) significantly covaried with the female propensity to explore the open arms of the elevated plus-maze, as observed before the onset of the cohabitation; (3) accounted for a substantial proportion of the defensive-submissive response of the female; (4) prolonged floating was associated with less sustained defensive-submissive behaviors; and (5) the extent of which was dampened by the anxious disposition of the female (moderation shown by a significant product-term). The model fit indices are presented (χ^2^, chi-square; CFI, comparative fit index; RMSEA, root mean square error of approximation). Path strengths are shown with their statistical significance (^*^p < 0.05; ^**^p < 0.01; ^***^p < 0.001) and are represented by an incremental line thickness, as shown in the figure. **(C)** Focus on the representation of the moderation of the outcome by anxiety. Although, defensive-submissive behaviors are equivalent at lower aggression levels and they overall increase when faced with a more aggressive partner, this increase is dampened by pre-existing female anxious-like temperament (*less* open arm exploration in the elevated plus-maze, EPM).

The structural equation modeling analysis teased apart some additional, putatively causal behavioral relationships (Figure 4). We sought to verify whether females' anxious temperament affected coping abilities in the face of social adversity (Figure 4A). As shown in Figure 4B, in addition to showing that correlational effects were maintained, such as the predictive ability of male partner aggression on increased defensive behavior (β = 0.67, *p* < 0.001) and subsequent floating behavior (β = 0.44, *p* < 0.05), as well as the covariation of male partner aggression with elevated plus-maze anxiety (φ = −0.36, *p* < 0.05), effects of anxiety on female defensive-submissive behavior and, in turn, effects of female defensive behavior on floating behavior emerged. While the extent of pre-cohabitation open arm exploration, i.e., low anxiety (1) marginally predicted a lower propensity for extensive floating (β = −0.27, *p* = 0.08), (2) it significantly contributed to the extent of the defensive-submissive behavior (β = 0.40, *p* < 0.001), (3) which in turn buffered against subsequent floating behavior (β = −0.37, *p* < 0.05). A differential defensive-submissive response to partner aggression that depended on female anxious temperament is revealed by the significant interaction term (β = 0.32, *p* < 0.01). This moderation portion of the model is graphically presented in Figure 4C, showing that the percentage of time in the open arms significantly dampened (or vice versa, low anxiety-like behavior amplified) the extent of defensive-submissive behavior elicited by partner aggression.

### Regional gene expression patterns associated with behavioral vulnerability to depressive-like symptoms

**Figure 5:**
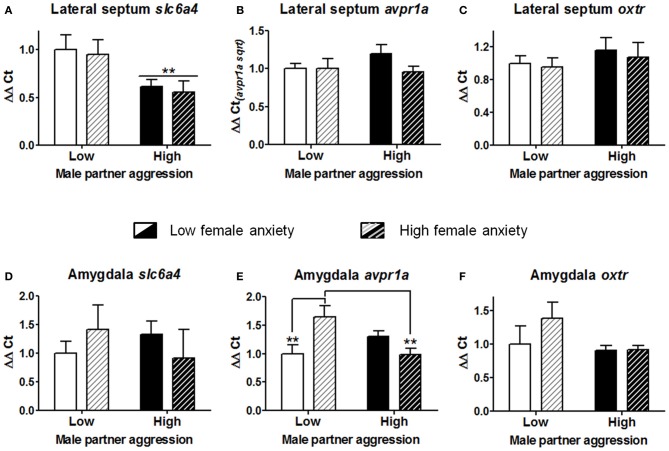
**Gene expression analyses of the serotonin transporter (*slc6a4*), vasopressin receptor 1a (*avpr1a*), and oxytocin receptor (*oxtr*) for the lateral septum (A–C) and the amygdala (D–F).** Cohabitation with an aggressive male partner reduced the expression of *slc6a4* in the lateral septum of the females. In the amygdala, partner aggression and female anxiety-like predisposition interacted to determine the *avpr1a* expression. Females that had exhibited lower exploration of the open arms in the elevated plus-maze (i.e., “high” vs. “low” anxiety) exhibited a higher level of expression of this gene, yet this augmentation was blunted with sustained partner aggression. In contrast, no significant alterations were observed for *oxtr* in these regions. The mean ± standard error of the mean are presented. Sqrt, square root.

In order to examine patterns of gene expression associated with the effects of male partner aggression and female anxiety-like behavior, subgroups were produced based on a median split for each variable [“low” vs. “high” anxiety-like behavior, mean percent (±S.E.M.) = 23.9(±1.8) and 7.0(±1.7), *t*_(22)_ = 6.8, *p* < 0.001]; and male partner aggression, respectively 3.6(±0.5) and 11.3(±1.7), *t*_(22)_ = −4.3, *p* < 0.001; *n* = 5–6 for each of the four subgroup combinations. As shown in Figure 5A, partner aggression led to a significant reduction of *slc6a4* expression in the lateral septum (Two-Way ANOVA on regional gene expression *F*_(1, 19)_ = 8.4, *p* < 0.01). In contrast, there were no main effects of partner aggression on either *avpr1a* or *oxtr* expression in the lateral septum (all *p* > 0.25; for amygdala, all *p* > 0.13, respectively Figures 5B,C). There was no main effect of anxiety-like behavior on any of the genes examined in the lateral septum (Figures 5A–C; all *p* > 0.26) or the amygdala (Figures 5D–F; all *p* > 0.27). Anxiety and male partner aggression interacted in one region, the amygdala, for only *avpr1a* expression (Two-Way ANOVA on regional gene expression, *F*_(1, 17)_ = 11.7, *p* < 0.005; both other genes, *p* > 0.14; for lateral septum, all *p* > 0.26). More specifically, as shown in Figure 5E, high anxiety females exhibited augmented *avpr1a* expression in the amygdala when partner aggression was relatively low (*p* < 0.01), and while partner aggression reduced the expression of this gene in high anxiety females (*p* < 0.005), in low anxiety females, *avpr1a* expression was not affected by partner aggression (other comparisons, *p* > 0.11).

## Discussion

Intimate partner violence is often associated with depressive outcomes (Beydoun et al., 2012), and the present study sought to uncover behaviors and neural markers that are associated with resilience to partner aggression. Using a rodent model of intimate partner violence and combining behavioral data and modeling (Castro et al., 2010), the current study revealed that individual differences in anxiety-like behavior were related to the outcome of partner aggression on ensuing depressive-like symptoms by dampening defensive-submissive coping behaviors. Furthermore, when investigating potential alterations of relevant genes in brain regions that are essential for the regulation of anxiety and social behaviors (i.e., the lateral septum and the amygdala), we found that while male partner aggression led to reduced expression of the serotonin transporter *slc6a4* in the lateral septum regardless of female anxiety, male partner aggression led to reduced vasopressin receptor 1a (*avpr1a*) expression in the amygdala only in high anxiety females. These findings identify the lateral septum as a brain region that, regardless of female anxiety is associated with vulnerability to depressive-like symptoms elicited by aggression exposure, as indicated by the reduced expression of a key gene in the serotonergic pathway. Importantly, our results indicate that anxious temperament can influence the outcome of aggressive cohabitation. The vulnerability of this at-risk population to depressive-like symptoms was found to be associated with alterations in amygdala vasopressinergic signaling.

The results of the modeling analysis imply that females that are less avoidant of the open arm in the elevated plus-maze subsequently behave in a such way that facilitates their subordination to an aggressive male partner, as evidenced by less male partner aggression and more defensive-submission (freezing or supine posture under the male). Such “agreeable,” “complementary” behavior (respectively, Moskowitz, 1994; Tiedens and Fragale, 2003) may mitigate the risks of living with an aggressive partner, and buffer from future depressive-like symptoms. The development of subordination appears in this case to be an adaptive strategy, and with respect to “proactive vs. reactive”/“hawk vs. dove” strategic coping patterns, these females may present characteristics of the latter: low risk-taking, non-aggressive, and cautious yet thorough in their environment appraisal (Koolhaas et al., 1999; Korte et al., 2005).

The present reciprocal partner influence of the behaviors of both the perpetrator and victim in our model is in line with clinical findings, further supporting the view that both partners need to be taken into account to understand a relationship and ultimately to improve prevention and treatment of psychopathology (Moffitt et al., 2001). A benefit of the rat model described here is the absence of assortative mating, which is a common issue in human studies and a potential confounder for the understanding of causal events in this dyadic relationship (Rhule-Louie and McMahon, 2007).

The association reported here with floating behavior in the forced swim paradigm may be particularly relevant to understanding vulnerability to the development of depressive-like symptomatology following intimate partner violence, particularly in terms of learned helplessness. Although, there is conflicting evidence on the association of disturbances of affect, such as depression and anxiety, with ensuing victimization [positive relationship; Kim and Capaldi, 2004; Amar and Gennaro, 2005; Lehrer et al., 2006; but no association in Raiford et al. (2007), Fergusson et al. (2008)], learned helplessness may mediate the relationship between violence and symptoms of both depression and post-traumatic disorder (Bargai et al., 2007).

Depression, conditioned defeat and learned helplessness have been associated with dorsal raphe function (Maier and Watkins, 2005; Goswami et al., 2010; Hammack et al., 2012). As there are direct dorsal raphe serotonergic projections to the lateral septum (Steinbusch, 1981; Risold and Swanson, 1997), a reduction in the serotonergic signaling regulator *slc6a4* (not in *maoa*, data not shown) in this region may be related to the disturbances that we previously reported for this neurotransmitter system in the dorsal raphe of females exposed to aggressive males (Cordero et al., 2012). In that previous study, females cohabitated either with a control or an aggressive male. Ten weeks after the cohabitation, dorsal raphe subregions were differentially affected, with females exhibiting reduced levels of serotonergic cell activation at baseline (ventrolateral) but increased levels upon exposure to an unfamiliar male (dorsal and caudal; Cordero et al., 2012), respectively previously associated with either anti-depressant or -panic properties, vs. anxiogenesis, as recently described in a review of the literatures of human imaging and post-mortem analyses as well as that of animal models (imaging, pharmacological, and lesion work on anxiety-, panic-, and depressive-like behavior, as respectively interpreted from the consequences of e.g., social or non-social inescapable stress, hypercapnia, and forced swim test consequences; Hale et al., 2012). Here, of particular interest is the reduced baseline activation in the ventrolateral subregion, one associated with anti-depressant properties. Lateral septum serotonin has also been proposed to be protective against depression (Sheehan et al., 2004), and it receives the majority of its dorsal raphe inputs from the ventrolateral subregion (Kanno et al., 2008).

The serotonin transporter plays an important role in emotion and social relations [reviewed by Canli and Lesch (2007)], and interestingly, a gene variant conferring low *slc6a4* function was reported to increase depression resulting from abuse in pregnant women (Scheid et al., 2007). The current finding of reduced serotonin transporter expression in the lateral septum of females subjected to increased aggression is consistent with the proposal [in review by Sheehan et al. (2004)] that chronic stress would reduce lateral septum activity, which could occur via increased serotonin or blunting of its reuptake. Such modulation of serotonin in the lateral septum would lead to its inhibition, and lower activity of the lateral septum has been associated with increased fear and learned helplessness-like behavior (Sheehan et al., 2004).

Altogether, these observations indicate that this system would be a prime candidate for the alleviation of the psychopathological symptoms associated with intimate partner violence. Among the potential mechanisms for the sustained changes found in our study in *slc6a4* expression could be epigenetic changes that are induced by exposure to aggression. Methylation of *slc6a4* from peripheral blood can yield reduced mRNA expression (Philibert et al., 2007) and reduced *in vivo* brain serotonin synthesis (Wang et al., 2012), in association with the affective outcome of traumatic events. Promoter methylation levels of the *slc6a4* gene may either protect or confer vulnerability to unresolved loss or related post-traumatic stress disorder, according to genetic variants at different sites (respectively, Van IJzendoorn et al., 2010; Koenen et al., 2011). Finally, although the administration of the serotonin precursor tryptophan has been found to increase agreeableness in men, it is less consistently so in women (aan het Rot et al., 2006; Young et al., 2007). Informing this gender discrepancy the present results may suggest for women the consideration of the vasopressinergic system, in association with anxious temperament.

A role for anxiety in modulating the engagement of the amygdala with social adversity would be consistent with evidence in mouse lines exhibiting anxiety differences, where higher anxiety was associated with enhanced social avoidance after repeated social defeat (Savignac et al., 2011). While serotonin transporter gene variants conferring reduced function have been associated with amygdala basal hyperactivity and hyper-reactivity to perceived threat in males and females (Hariri et al., 2002; Canli et al., 2006), no differences were observed in the expression of this gene in the amygdala in the present study. In contrast, alterations were observed for the *avpr1a* gene. Notably, the vasopressinergic system has also been associated with depression, as well as anxiety. Vasopressin activates the hypothalamus-pituitary-adrenal axis and can exert anxiogenic properties [reviewed in Engelmann et al. (2004)]. Strikingly, vasopressin functional effects appear to depend on the socio-emotional context. Therefore, although peripheral vasopressin was associated with positive social couple relations in a non-depressed sample (De Winter et al., 2012), in depressed samples, it was found to positively correlate with the extent of the disorder, and in particular with the anxious vs. non-anxious depression subtype (Van Londen et al., 1997; De Winter et al., 2003).

In the amygdala, *avpr1a* is present mainly in the central nucleus [female and male voles, (Insel et al., 1994); male rats, (Veinante and Freund-Mercier, 1997)]. Anxiety and *avpr1a* expression in the amygdala have been associated in females. Lactating dams from rat lines bred for high anxiety exhibit more aggressive behavior than those bred for low anxiety. The aggression is associated with greater release of vasopressin in the central amygdala nucleus, which is dependent on *avpr1a* (Bosch and Neumann, 2010). It should also be noted that in addition to aggression, alterations in central amygdala vasopressin receptor 1a gene expression may be related to post-partum maternal behaviors in rats (Caughey et al., 2011) and humans (Bisceglia et al., 2012); however, any potential vasopressin effects on maternal behavior were found to be unrelated to the dam's anxiety in rats (Bosch and Neumann, 2008).

It should be noted that the lateral septum and the amygdala are interconnected (Risold and Swanson, 1997), may reciprocally modulate each other, and are both part of what has been termed a “social behavior neural network” (Newman, 1999). Our results suggest that such a putative regulatory loop may be disturbed in females that develop depressive-like symptoms following their experience with an aggressive partner *via* amygdala vasopressin signaling, particularly those with a more anxious temperament at baseline.

Therefore, the present findings provide potentially useful insight for the development of clinical intervention for trauma caused by intimate partner violence. These findings emphasize the importance of accounting for individual differences in temperament to uncover substrates of vulnerability to social adversity, which may prove useful in addressing variability in responses to psycho- and pharmacotherapy. Manipulations of the proposed targets in the amygdala and the lateral septum could help increase resilience and promote recovery from social trauma. Integrating this information into basic research and ultimately clinical practice may prove fruitful in evaluating treatment opportunities and improving translational success.

### Conflict of interest statement

The authors declare that the research was conducted in the absence of any commercial or financial relationships that could be construed as a potential conflict of interest.
